# Suboptimal self‐reported sleep efficiency and duration are associated with faster accumulation of brain amyloid beta in cognitively unimpaired older adults

**DOI:** 10.1002/dad2.12579

**Published:** 2024-04-22

**Authors:** Louise N. Pivac, Belinda M. Brown, Kelsey R. Sewell, James D. Doecke, Victor L. Villemagne, Vincent Doré, Michael Weinborn, Hamid R. Sohrabi, Samantha L. Gardener, Romola S. Bucks, Simon M. Laws, Kevin Taddei, Paul Maruff, Colin L. Masters, Christopher Rowe, Ralph N. Martins, Stephanie R. Rainey‐Smith

**Affiliations:** ^1^ Centre for Healthy Ageing, Health Futures Institute Murdoch University Murdoch Western Australia Australia; ^2^ Alzheimer's Research Australia, Sarich Neuroscience Research Institute Nedlands Western Australia Australia; ^3^ Australian E‐Health Research Centre, CSIRO Herston Queensland Australia; ^4^ Department of Psychiatry University of Pittsburgh Pittsburgh Pennsylvania USA; ^5^ Department of Molecular Imaging Austin Health Heidelberg Victoria Australia; ^6^ School of Psychological Science University of Western Australia Perth Western Australia Australia; ^7^ School of Medical and Health Sciences Edith Cowan University Joondalup Western Australia Australia; ^8^ School of Population and Global Health University of Western Australia Perth Western Australia Australia; ^9^ Centre for Precision Health Edith Cowan University Joondalup Western Australia Australia; ^10^ Collaborative Genomics and Translation Group Edith Cowan University Joondalup Western Australia Australia; ^11^ Curtin Medical School Curtin University Bentley Western Australia Australia; ^12^ Cogstate Ltd., Melbourne Melbourne Victoria Australia; ^13^ The Florey Institute of Neuroscience and Mental Health University of Melbourne Melbourne Victoria Australia; ^14^ Department of Biomedical Sciences Macquarie University Macquarie University Sydney New South Wales Australia

**Keywords:** AIBL study, Alzheimer's disease, amyloid, apolipoprotein E ε4, positron emission tomography, sleep

## Abstract

**INTRODUCTION:**

This study investigated whether self‐reported sleep quality is associated with brain amyloid beta (Aβ) accumulation.

**METHODS:**

Linear mixed effect model analyses were conducted for 189 cognitively unimpaired (CU) older adults (mean ± standard deviation 74.0 ± 6.2; 53.2% female), with baseline self‐reported sleep data, and positron emission tomography‐determined brain Aβ measured over a minimum of three time points (range 33.3–72.7 months). Analyses included random slopes and intercepts, interaction for apolipoprotein E (*APOE*) ε4 allele status, and time, adjusting for sex and baseline age.

**RESULTS:**

Sleep duration <6 hours, in *APOE* ε4 carriers, and sleep efficiency <65%, in the whole sample and *APOE* ε4 non‐carriers, is associated with faster accumulation of brain Aβ.

**DISCUSSION:**

These findings suggest a role for self‐reported suboptimal sleep efficiency and duration in the accumulation of Alzheimer's disease (AD) neuropathology in CU individuals. Additionally, poor sleep efficiency represents a potential route via which individuals at lower genetic risk may progress to preclinical AD.

**Highlights:**

- In cognitively unimpaired older adults self‐report sleep is associated with brain amyloid beta (Aβ) accumulation.
- Across sleep characteristics, this relationship differs by apolipoprotein E (*APOE*) genotype.
- Sleep duration <6 hours is associated with faster brain Aβ accumulation in *APOE* ε4 carriers.
- Sleep efficiency < 65% is associated with faster brain Aβ accumulation in *APOE* ε4 non‐carriers.
- Personalized sleep interventions should be studied for potential to slow Aβ accumulation.

## 1 BACKGROUND

There is increasing evidence of a bidirectional relationship between suboptimal sleep and brain amyloid beta (Aβ) burden, indicating that suboptimal sleep both results from and contributes to the accumulation of brain Aβ, a pathological hallmark of Alzheimer's disease (AD).^1^, ^2^, ^3^, ^4^, ^5^, ^6^, ^7^ While good quality sleep enhances brain health, suboptimal sleep has been proposed to lead to both the overproduction of brain Aβ, as a result of increased neuronal activity,^8^, ^9^, ^10^ and reduced Aβ clearance via the glymphatic system,^10^, ^11^, ^12^, ^13^ leading to the accumulation of toxic oligomers and, ultimately, the formation of brain Aβ plaques.^8^, ^9^, ^10^, ^11^, ^12^, ^14^, ^15^, ^16^, ^17^ While cognitive impairment and dementia due to AD pathology are typically diagnosed in later life, the accumulation of brain Aβ begins decades earlier while individuals are cognitively unimpaired (CU).^18^

Cross‐sectional studies of CU older adults, using brain Aβ positron emission tomography (PET), have reported associations between suboptimal night‐time sleep (including self‐reported sleep quality, sleep onset latency, and short sleep duration, and objectively measured sleep onset latency, sleep fragmentation, and sleep efficiency) and greater brain Aβ burden.^3^, ^6^, ^7^, ^19^, ^20^, ^21^, ^22^, ^23^, ^24^ However, only one longitudinal study has investigated the capacity of poor night‐time sleep characteristics to predict brain Aβ dynamics. The authors of this longitudinal study of CU older adults (*N* = 32) reported that objectively measured sleep efficiency and proportion of slow wave sleep, below 1 Hz, predicted the subsequent rate of brain Aβ accumulation.^4^ Characteristics of sleep quality may therefore be viewed as a prospective biomarker of brain Aβ accumulation while also representing a potentially modifiable risk factor for AD. However, to our knowledge, no study to date has investigated whether self‐reported sleep quality characteristics are associated with brain Aβ accumulation, a knowledge gap the current study addresses. The current study assesses self‐reported sleep, in CU older adults, via the Pittsburgh Sleep Quality Index (PSQI), a valid, reliable tool, frequently adopted in sleep research among a range of populations including older adults.^25^, ^26^, ^27^ CU older adults represent a group with intact cognition who are therefore better able to accurately self‐report sleep quality, and ultimately represent a potential target population for sleep improvement interventions aimed at preventing the onset of cognitive decline due to AD.

The apolipoprotein E (*APOE*) ε4 allele is a strong genetic risk factor for brain Aβ deposition, influencing Aβ clearance and aggregation.^28^, ^29^ In AD patients, *APOE* ε4 non‐carriage was found to be associated with greater deterioration in sleep quality with advancing age.^30^ Moreover, a small body of cross‐sectional research suggests that *APOE* genotype potentially interacts with sleep quality to determine risk of AD‐related pathological change (including Aβ pathology, tau pathology, and gray matter volume), though the evidence is inconsistent, potentially due to the heterogeneity of study samples and methodologies.^3^, ^31^, ^32^, ^33^ The impact of *APOE* ε4 on the relationship between sleep and brain Aβ accumulation has not been investigated; the current study addresses this knowledge gap.

Thus, the current study sought to evaluate whether self‐reported sleep quality characteristics are associated with subsequent brain Aβ accumulation, assessed over a minimum of 3 years, in a highly characterized sample of CU older adults, and to investigate whether the results differ based on *APOE* ε4 allele carriage. The sample was drawn from the Australian Imaging, Biomarkers and Lifestyle (AIBL) Study of Ageing. We hypothesized a faster increase in brain Aβ burden over time in poor sleepers, with the effect magnified in *APOE* ε4 carriers.

## 2 METHODS

### 2.1 Study population

**FIGURE 1:**
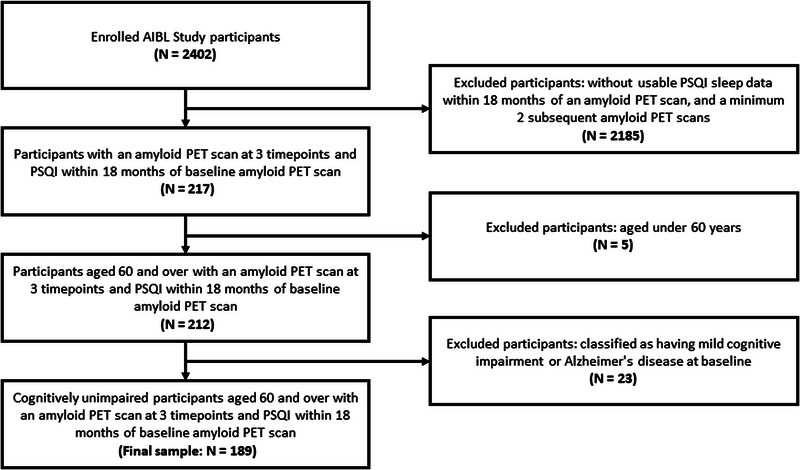
Participant selection flowchart. AIBL, Australian Imaging, Biomarkers and Lifestyle Study of Ageing; PET, positron emission tomography; PSQI, Pittsburgh Sleep Quality Index.

This longitudinal investigation (up to 6 years’ follow‐up) used data from 189 participants drawn from the AIBL Study of Ageing. Included participants (see Figure 1) were aged ≥ 60 years at study enrollment, were classified as CU at baseline, and underwent brain Aβ PET imaging at baseline then at a minimum two further AIBL timepoints (AIBL assessments occur at 18‐month intervals; for the current sample, median 3.0 PET scans, median absolute deviation 0.0). Included individuals had also completed the PSQI^34^ within 18 months of their baseline PET scan. Data were collected from July 2012 to July 2019. The AIBL Study, including recruitment methods, screening criteria, and diagnostic classification procedures, has been described in detail elsewhere.^35^, ^36^ AIBL is approved by the institutional ethics committees of Austin Health, St. Vincent's Health, Hollywood Private Hospital (now Ramsay Health Care), Murdoch University, and Edith Cowan University. All individuals provided written informed consent prior to undergoing any study procedures or assessments.

**RESEARCH IN CONTEXT**

1. **Systematic review**: To identify longitudinal studies considering sleep and brain amyloid beta (Aβ) dynamics, a standard literature search was conducted (e.g., PubMed). Cross‐sectional studies report the relationship between sleep quality and brain Aβ burden. However, only one longitudinal study was identified. No previous study has included a large sex‐balanced sample, used a self‐report sleep measure, or considered the role of apolipoprotein E (*APOE*) ε4 carriage.
2. **Interpretation**: Sleep duration <6 hours was associated with faster brain Aβ accumulation in *APOE* ε4 carriers while sleep efficiency < 65% was associated with faster brain Aβ accumulation in the whole sample and in *APOE* ε4 non‐carriers.
3. **Future directions**: To better understand sleep's role in brain Aβ accumulation, and the point at which divergence occurs between *APOE* ε4 non‐carriers and carriers, longitudinal research with mid‐life cohorts is required. Additionally, intervention studies are essential to determine whether improving suboptimal sleep would slow the rate of brain Aβ accumulation.

### 2.2 Sleep measures

The PSQI, a 19‐item, valid, reliable measure of self‐reported sleep quality over the past month, frequently adopted in aging research, was used to derive self‐report sleep characteristics for analysis.^25^ PSQI items are grouped into seven sleep quality component scores which, when summed, produce a PSQI global score, with poor sleep indicated by a global score >5.^25^ As the current study is the first, to our knowledge, to consider the relationship between self‐reported night‐time sleep quality and brain Aβ accumulation, all night‐time sleep characteristics measured by the PSQI have been included. Specifically, in the current study, the following sleep characteristics were investigated for associations with accumulation of brain Aβ: sleep duration (hours and category), sleep onset latency (time to fall asleep; minutes and category), sleep efficiency (time in bed spent asleep; percentage and category), sleep disturbance (category), sleep quality (categorical response to question “how would you rate your overall sleep quality?”) and global PSQI score (a continuous measure of overall sleep quality). Determination of sleep duration categories was informed by The National Sleep Foundation Guidelines.^37^ As our participant group was aged 60 to 80 years, reference to both the recommendations for adults (26–64 years) and older adults (≥ 65 years) was considered, and sleep duration was categorized as <6 hours (short), 6 to 8 hours (optimal), or 8 hours (long). In the absence of National Sleep Foundation Guidelines for sleep efficiency and sleep onset latency, creation of categories for these variables was guided by the PSQI scoring instructions, with sleep efficiency categorized as > 85% (optimal), 65% to 85% (poor), and < 65% (very poor), while sleep onset latency was categorized as <30 minutes (optimal), 30 to 59 minutes (long), and 60+ minutes (very long). Further description of categorical variables, reference groups, and response frequencies can be found in Table S1 in supporting information.

### 2.3 Brain imaging

Brain Aβ PET imaging using one of five Aβ‐binding tracers (^11^C‐Pittsburgh compound B, ^18^F‐flutemetamol, ^18^F‐florbetapir, ^18^F‐Florbetaben, or ^18^F‐NAV4694), was conducted per standard protocols, as detailed elsewhere.^38^, ^39^, ^40^ The CapAIBL quantification algorithm was used to calculate a Centiloid (CL) value for each PET image, providing a single continuous variable representing brain Aβ burden.^41^ For each participant included in the current analysis, brain Aβ PET imaging was conducted at a minimum three AIBL timepoints.

### 2.4 *APOE* genotyping

Participants donated fasted blood samples, from which DNA was extracted and analyzed to determine *APOE* genotype, per standard protocols described elsewhere.^36^ *APOE* ε4 allele carrier status (carrier or non‐carrier) was included in data analysis.

### 2.5 Statistical analysis

Statistical analyses were carried out using R version 4.1.0. Baseline demographics, brain imaging, and sleep characteristics were determined, and between‐group differences assessed using independent samples *t* tests (continuous variables), chi‐square (χ^2^) analysis (categorical variables), or the Kruskal–Wallis test (ordinal variable and median difference; Table 1). A linear mixed effects model (LMM), including random slopes and intercepts, interaction for *APOE* ε4 allele status, and time, adjusting for sex and baseline age (confirmed by model fit analysis as appropriate), was used to model change in brain Aβ over a minimum of three PET scan collections, as predicted by each sleep parameter.

**TABLE 1:** Baseline demographic, brain imaging, and sleep characteristics for the cognitively unimpaired sample who completed the PSQI at baseline and underwent Aβ PET imaging at a minimum three AIBL study timepoints.

	Whole sample (*N *= 189)	*APOE ε*4 non‐carriers (*N* = 137)	*APOE ε*4 carriers (*N* = 52)	*P* value^a^
**Demographics**				
Age, years	73.7 ± 5.6	73.7 ± 5.7	73.7 ± 5.5	0.967
Female, *n* (%)	99 (52.4)	72 (52.6)	27 (51.9)	0.938
Education >12 years, *n* (%)	102 (54.0)	77 (56.2)	25 (48.1)	0.317
MMSE	29.0(1.1)	29.0 (1.1)	29.0 (1.2)	0.842
GDS	1.2 (1.4)	1.2 (1.3)	1.2(1.7)	0.925
BMI^b^	26.5 (4.2)	26.7 (4.2)	25.8 (4.2)	0.153
**Brain imaging characteristics**				
Number of amyloid PET scans (range)	3.3 (3 ‐ 5)	3.3 (3 ‐ 5)	3.4 (3 ‐ 5)	0.256
Follow up period^c^, median months (MAD)	52.4 (1.1)	51.3 (1.2)	54.2 (0.8)	0.079
Baseline brain Aβ, Centiloid	19.1 (30.9)	13.3 (26.9)	34.7 (35.5)	**<0.001**
High brain Aβ burden, *n* (%)^d^	60 (31.7)	32 (23.4)	28 (53.8)	**<0.001**
**Sleep characteristics**				
Sleep duration, hours	6.9 (1.2)	6.7 (1.1)	7.1 (1.3)	**0.021**
Sleep efficiency, %	80.2 (13.2)	79.1 (12.8)	84.0 (14.1)	0.079
Sleep onset latency, minutes	20.5 (20.9)	21.4 (22.4)	18.0 (16.2)	0.316
PSQI global score	6.1 (3.6)	6.3 (3.5)	5.5 (3.8)	0.174
Good sleepers^e^, *n* (%)	98 (51.9)	64 (46.7)	30 (62.5)	**0.022**
Sleep medication use^f^	0.5 (0.9)	0.5 (0.9)	0.5 (1.0)	0.970
PSQI to PET scan, months	8.8 (6.4)	8.6 (6.4)	9.2 (5.7)	0.549
**Baseline brain Aβ, Centiloid, by sleep characteristic**				
Sleep duration < 6 hours	25.8 (27.1)	17.5 (23.5)	54.8 (17.3)	**0.001**
Sleep duration 6–8 hours	19.7 (32.6)	14.0 (28.5)	34.5 (38.0)	**<0.001**
Sleep duration > 8 hours	4.8 (14.9)	−0.7 (8.6)	15.8 (14.5)	**0.022**
Sleep efficiency < 65%	27.7 (27.8)	20.6 (24.5)	46.0 (25.2)	**0.030**
Sleep efficiency 65%–85%	17.5 (31.8)	11.8 (26.2)	37.6 (40.9)	**0.001**
Sleep efficiency > 85%	18.2 (31.2)	12.6 (28.5)	29.4 (33.9)	**0.024**
Sleep onset latency < 30 minutes	18.7 (32.2)	12.5 (28.6)	33.2 (35.8)	**<0.001**
Sleep onset latency 30–59 minutes	18.4 (27.7)	13.6 (23.5))	32.7 (35.4)	0.074
Sleep onset latency 60+ minutes	24.3 (27.5)	17.4 (21.5)	72.4 (3.5)	**0.003**
“Very good” sleep quality	20.1 (29.3)	16.8 (30.3)	27.1 (26.5)	0.224
“Fairly good” sleep quality	17.8 (32.9)	11.4 (27.5)	36.1 (40.1)	**<0.001**
“Bad” sleep quality	22.0 (27.2).	13.5 (16.9)	48.6 (36.9)	**0.001**

*Notes*: Unless otherwise noted, all values are mean ± SD. Bold values indicate significance at P < 0.05.

Abbreviations: Aβ, amyloid beta; AIBL, Australian Imaging, Biomarkers and Lifestyle Study; *APOE*, apolipoprotein E; BMI, body mass index; GDS, Geriatric Depression Scale; MAD, median absolute deviation; MMSE, Mini‐Mental State Examination; PET, positron emission tomograpy; PSQI, Pittsburgh Sleep Quality Index; SD, standard deviation.

^a^ *p*‐values for independent sample *t* tests, χ^2^ tests or Kruskal–Wallis tests comparing *APOE* ε4 non‐carriers and carriers.

^b^ BMI was calculated as weight in kilograms divided by height in meters squared.

^c^ Baseline to final PET scan interval.

^d^ Threshold for high brain Aβ burden is ≥ 20 Centiloid.

^e^ Good sleepers were defined based on a PSQI global score of ≤ 5.

^f^ PSQI sleep medication component score: “During the past month, how often have you taken medicine (prescribed or ‘over the counter’) to help you sleep?”

Given the previous findings that brain Aβ accumulation begins ~15 years earlier for *APOE* ε4 carriers^42^ compared to non‐carriers, we tested the LMM framework for both a two‐way interaction with *APOE* ε4 carriage and time, and a three‐way interaction among sleep, *APOE* ε4 carriage, and time, with brain Aβ the dependent variable. A significant interaction for *APOE* ε4 carriage and time (*P* = 0.001) and a trend toward significance for the three‐way interaction (moderate/severe sleep disturbance, *P *= 0.046, sleep duration category < 6 hours, *P* = 0.085, and sleep efficiency category 65%–85%, *P *= 0.070) confirmed modeling stratified for *APOE* ε4 carriage was warranted. This model included random slopes, random intercepts, interaction effects for time, and *APOE* ε4 allele carriage, to model against the 95% Winsorized CL value. Covariates excluded through this process were an age–sex interaction, cardiovascular risk score, body mass index (BMI), depressive symptomology (Geriatric Depression Scale [GDS]), education category (≤ 12 years/> 12 years) and the use of sleep‐related medications. Statistical significance was set at *P* < 0.05, with a Bonferroni‐corrected threshold of 0.008 (0.05/6) applied to account for multiple comparisons. Further statistical methodology can be found in the Statistical Methods section in supporting information.

## 3 RESULTS

Baseline demographic, brain imaging, and sleep characteristics for AIBL participants (*N* = 189) included in the current study are presented in Table 1. There were no significant differences for demographic factors between *APOE* ε4 non‐carriers and carriers. As expected, compared to non‐carriers, *APOE* ε4 carriers had higher brain Aβ at baseline (mean difference = 21.4 CL, *P* < 0.001), and a greater proportion (% difference = 30.4) of individuals with high brain Aβ burden (CL ≥ 20; *P* < 0.001). *APOE* ε4 non‐carriers reported shorter sleep duration compared to *APOE* ε4 carriers (mean difference = 24 minutes, *P *= 0.021), and when participants were categorized as either “good” or “poor” sleepers (poor sleeper = PSQI global score > 5), there was a greater proportion (18.9%) of “poor” sleepers (*P* = 0.022) in the *APOE* ε4 non‐carrier group compared to *APOE* ε4 carriers.

### 3.1 Sleep duration and rate of brain Aβ accumulation

**FIGURE 2:**
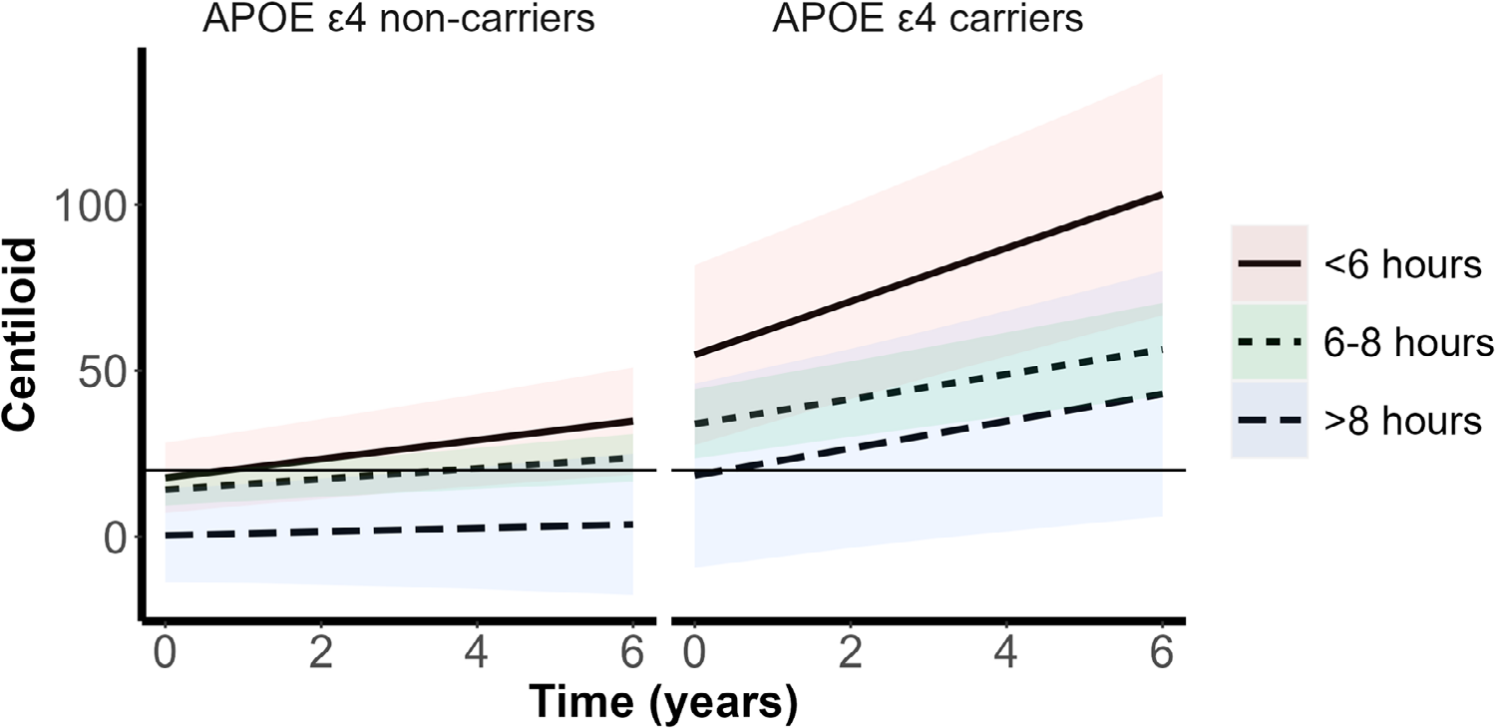
Plots for the relationship between baseline sleep duration category and the trajectory of brain Aβ burden, in cognitively unimpaired *APOE* ε4 allele non‐carriers (left) and carriers (right). Brain Aβ burden is presented as Centiloid value (95% Winsorized). Sleep duration category was derived from PSQI Question 4, self‐report hours of actual sleep, with < 6 hours of sleep duration categorized as “short", 6 to 8 hours of sleep duration as “normal", and >8  hours of sleep duration as “long". The horizontal line, at a Centiloid value of 20, represents the threshold for high brain Aβ burden. Colored bands represent 95% confidence intervals. Sleep duration category < 6 hours was associated with greater brain Aβ burden at baseline and a steeper slope of brain Aβ accumulation in *APOE* ε4 allele carriers. Comparison of the baseline Centiloid value (*y* intercept) and final Centiloid value for *APOE* ε4 allele carriers with < 6 hours of sleep and those with 6 to 8 hours of sleep indicated that those with < 6 hours of sleep accumulated ~ 23% more brain Aβ over time. Aβ, amyloid beta; *APOE*, apolipoprotein E; PSQI, Pittsburgh Sleep Quality Index.

As presented in Table 2, for the whole cohort (β = −0.67 ± 0.26, *P* = 0.012) and for *APOE* ε4 non‐carriers (β = −0.66 ± 0.25, *P* = 0.010), longer total sleep time was nominally associated with slower brain Aβ accumulation over time, after Bonferroni correction, whereby for each hour of sleep, 0.67 fewer CL units of amyloid were accumulated per year. At baseline, longer sleep duration in *APOE* ε4 carriers was nominally associated with lower brain Aβ burden (β = −9.17 ± 3.57, *P* = 0.011). To determine whether the relationship between sleep duration and brain Aβ accumulation reflected a U‐shaped curve, as is reported for sleep and cognition, we analyzed a categorical sleep duration variable (see Table 2, Table S2 in supporting information, and Figure 2). For *APOE* ε4 carriers (β = 4.33 ± 1.58, *P* = 0.007), but not for the whole cohort or *APOE* ε4 non‐carriers, baseline sleep duration of < 6 hours was associated with a faster rate of brain Aβ accumulation compared to optimal sleep duration of 6 to 8 hours (see Table 2 and Table S2). Figure 2 demonstrates that *APOE* ε4 carriers with < 6 hours of sleep accumulated approximately 23% more brain Aβ over a 6‐year period than ε4 carriers with 6 to 8 hours of sleep. Inspection of the *y* intercepts of Figure 2 show the absence of a U‐shaped curve for the relationship between sleep duration and brain Aβ. Instead, this figure shows that in both *APOE* ε4 non‐carriers and carriers, those with > 8 hours of sleep had a lower baseline brain Aβ burden compared to groups with shorter sleep duration, while those with < 6 hours of sleep presented at baseline with higher brain Aβ load, though these main effects were not significant.

**TABLE 2:** Results of linear mixed models examining the associations among baseline total sleep time, sleep duration category, sleep efficiency, sleep efficiency category, and brain Aβ burden over time.

	Whole sample (*N* = 189)		*APOE ε*4 non‐carriers (*N = *137)		*APOE ε*4 carriers (*N = *52)	
Models/Predictors	β	*P* value	β	*P* value	β	*P* value
Total sleep time (hours) x Time	−0.67 ± 0.26	**0.012**	−0.66 ± 0.25	**0.010**	−0.51 ± 0.41	0.218
< 6 hours sleep duration^a^ x Time	1.29 ± 0.84	0.124	1.25 ± 0.82	0.130	4.33 ± 1.58	**0.007^#^**
Sleep efficiency (%) x Time	−0.05 ± 0.02	**0.025**	−0.05 ± 0.02	**0.021**	−0.02 ± 0.04	0.534
<65% sleep efficiency^b^ x Time	2.91 ± 0.93	**0.002^#^**	2.86 ± 0.89	**0.001^#^**	1.32 ± 1.60	0.410

*Notes*: Models include PSQI‐derived sleep measure, *APOE* ε4 allele carrier status (±), time from baseline PET scan until final PET scan, baseline age, and sex as main effects, and three‐way sleep x *APOE* ε4 status x Centiloid interaction. Beta coefficients (β) ± SE from the LMM are shown, with bold indicating nominal significance (*P* < 0.05) and ^#^indicating significance after Bonferroni correction (*P* < 0.008). Brain Aβ burden in Centiloid units is derived from PET scans, conducted at a minimum three time points, with a median follow‐up period of 51.55 months (MAD = 13.01). Sleep duration category, derived from PSQI Question 4, self‐report hours of actual sleep, with < 6 hours of sleep duration categorized as “short,” 6–8 hours of sleep duration as “normal,” and > 8 hours of sleep duration as “long.” Sleep efficiency category, derived from PSQI‐determined sleep efficiency, which represents the percentage of time in bed spent asleep, with < 65% sleep efficiency categorized as “very poor,” 65% to 85% sleep efficiency as “suboptimal,” and > 85% sleep efficiency as “optimal.”

Abbreviations: Aβ, amyloid beta; *APOE*, apolipoprotein E gene; LMM, linear mixed model; MAD, median absolute deviation; PET, positron emission tomography; PSQI, Pittsburgh Sleep Quality Index; SE, standard error.

^a^Compared to 6–8 hours of (“normal”) sleep duration.

^b^ Compared to > 85% (“optimal”) sleep efficiency.

### 3.2 Sleep efficiency and rate of brain Aβ accumulation

**FIGURE 3:**
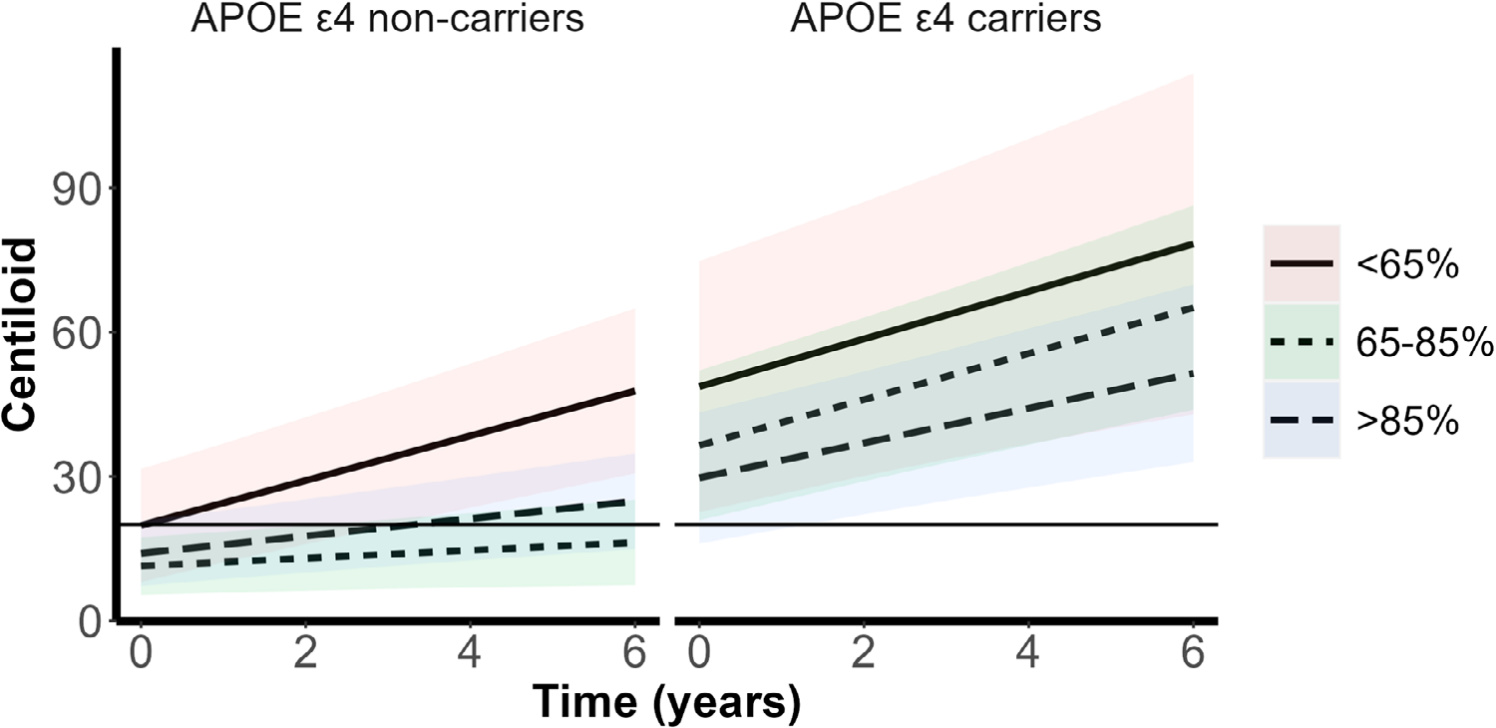
Plots for the relationship between baseline sleep efficiency category and the trajectory of brain Aβ burden, in cognitively unimpaired *APOE* ε4 allele non‐carriers (left) and carriers (right). Brain Aβ burden is presented as Centiloid value (95% Winsorized). Sleep efficiency category was derived from PSQI‐determined sleep efficiency, which represents the percentage of time in bed spent asleep, with < 65% sleep efficiency categorized as “very poor", 65% to 85% sleep efficiency as “suboptimal", and > 85% sleep efficiency as “optimal". The horizontal line, at a Centiloid value of 20, represents the threshold for high brain Aβ burden. Colored bands represent 95% confidence intervals. Sleep efficiency category < 65% was associated with greater brain Aβ burden at baseline and a steeper slope of brain Aβ accumulation in *APOE* ε4 allele non‐carriers. Comparison of the baseline Centiloid value (*y* intercept) and final Centiloid value for *APOE* ε4 non‐carriers with < 65% sleep efficiency and those with > 85% sleep efficiency indicated that those with < 65% sleep efficiency accumulated ~ 57% more brain Aβ over time. Aβ, amyloid beta; *APOE*, apolipoprotein E; PSQI, Pittsburgh Sleep Quality Index.

As shown in Table 2, after Bonferroni correction, higher baseline sleep efficiency was nominally associated with slower brain Aβ accumulation over time, within the whole cohort (β = −0.05 ± 0.02, *P* = 0.025), and in *APOE* ε4 non‐carriers (β = −0.05 ± 0.02, *P* = 0.021), but not in *APOE* ε4 carriers. Analysis of categorical sleep efficiency (see Table 2 and Figure 3) revealed that baseline sleep efficiency of < 65% was associated with faster brain Aβ accumulation over time relative to those with sleep efficiency > 85%, in the whole cohort (β = 2.91 ± 0.93, *P* = 0.002), and in *APOE* ε4 non‐carriers (β = 2.86 ± 0.89, *P* = 0.001), but not in *APOE* ε4 carriers (see Table S2). Figure 3 shows that those *APOE* ε4 non‐carriers with sleep efficiency < 65% accumulated ~ 57% more brain Aβ over 6 years compared to *APOE* ε4 non‐carriers with sleep efficiency of > 85%. Additionally, visual inspection of the *y* intercepts in Figure 3 reveals that, at baseline, both *APOE* ε4 non‐carriers and carriers with < 65% sleep efficiency had a higher brain Aβ burden than those with sleep efficiency of 65% to 85%, or > 85%, though these main effects were not significant.

### 3.3 Self‐reported sleep quality category and rate of brain Aβ accumulation

For *APOE* ε4 carriers (β = 3.42 ± 1.64, *P* = 0.038), but not for the whole cohort or *APOE* ε4 non‐carriers, self‐reported “poor” sleep quality was nominally associated with a faster rate of brain Aβ accumulation compared to those reporting “very good” sleep quality (see Table S2 and Figure S1 in supporting information), after Bonferroni correction. Moreover, the *y* intercepts of Figure S1 suggest that *APOE* ε4 carriers reporting “poor” sleep quality have greater baseline brain Aβ burden compared to *APOE* ε4 carriers with “very good” or “fairly good” self‐reported sleep quality, though these main effects were not significant.

### 3.4 Sleep onset latency and rate of brain Aβ accumulation

Analysis of sleep onset latency variables, both continuous and categorical, revealed no significant relationships, for any sub‐group comparison, with respect to rate of brain Aβ accumulation (see Table S2 and Figure S2 in supporting information). However, Figure S2 clearly shows that *APOE* ε4 carriers with sleep onset latency of ≥ 60 minutes had higher brain Aβ levels at both baseline, and over time, compared to *APOE* ε4 carriers with sleep onset latency < 60 minutes, though the rate of brain Aβ accumulation did not differ. The main effect for sleep onset latency of ≥ 60 minutes (compared to < 30 minutes) was approaching nominal significance (β = 46.77 ± 24.61, *P* = 0.059).

### 3.5 Sleep disturbance score and rate of brain Aβ accumulation

After Bonferroni correction for multiple comparisons, a nominally significant three‐way interaction was observed among moderate/severe sleep disturbance, *APOE* ε4 carriage, and time (β = 2.35 ± 1.17, *P* = 0.046), with respect to rate of brain Aβ accumulation. While a trend toward nominal significance was observed in *APOE* ε4 carriers with moderate/severe sleep disturbance (compared to mild sleep disturbance; β = 1.96 ± 1.06, *P* = 0.065), sleep disturbance score produced no significant results for any sub‐group with respect to rate of brain Aβ accumulation (see Table S2).

### 3.6 PSQI global score and rate of brain Aβ accumulation

Analyses of the PSQI global score (quantitative indicator of overall sleep quality based on the sum of PSQI component scores) produced no significant results, for any sub‐group, with respect to rate of brain Aβ accumulation (see Table S2).

## 4 DISCUSSION

The current study investigated the relationship between self‐reported sleep characteristics and rate of brain Aβ accumulation, over up to 6 years, in CU older adults. It is the first study to demonstrate an association between self‐reported night‐time sleep characteristics and the accumulation of brain Aβ measured by PET. After controlling for sex, and age, we found that both shorter sleep duration (< 6 hours) in *APOE* ε4 carriers, and poor sleep efficiency (< 65%) in the whole sample and in *APOE* ε4 non‐carriers, was associated with a faster rate of brain Aβ accumulation (compared to sleep duration of 6–8 hours and sleep efficiency of > 85%, respectively).

A longitudinal analysis conducted by Blackman et al.^43^ using PSQI component scores and cerebrospinal fluid (CSF) measures of Aβ did not find any relationship between short sleep duration and poor sleep efficiency, and changes in CSF Aβ over time. However, this study was of shorter duration (follow‐up 1.5 years ± 0.5) and > 80% of participants provided only one sample of CSF.^43^ Nevertheless, the authors did find that greater sleep disturbance was associated with faster decline in CSF Aβ42 levels (proposed to represent faster brain Aβ deposition).^43^ In the current study, no relationship between sleep disturbance and rate of brain Aβ accumulation was observed, although a trend toward significance was noted in *APOE* ε4 carriers.

While no prior longitudinal study has considered the relationship between self‐reported night‐time sleep quality and accumulation of brain Aβ, Carvalho et al.^44^ reported that individuals self‐reporting excessive daytime sleepiness (EDS) demonstrated faster accumulation of brain Aβ compared to those without EDS. This is consistent with Spira et al.,^45^ who reported that those with EDS were 2.75 times more likely to have high brain Aβ at follow‐up (15.7 years ± 3.4). While these studies did not investigate night‐time sleep duration or sleep efficiency, short and/or inefficient night‐time sleep may contribute to the EDS reported.

While past cross‐sectional analyses, using PET imaging, have reported a relationship between higher brain Aβ burden and both shorter self‐reported sleep duration^6^, ^21^ and poorer objectively measured sleep efficiency,^20^ our longitudinal analyses showed that sleep duration and sleep efficiency as continuous variables were not significantly associated with brain Aβ accumulation, after correction for multiple comparison, suggesting that this relationship is more complex. Indeed, it appears that thresholds of poor self‐reported sleep may be of greater utility in determining (1) who could benefit most from sleep improvement interventions in the context of slowing brain Aβ accumulation, and (2) that poorer self‐reported sleep (duration < 6 hours and/or efficiency < 65%) shows promise as a biomarker for subsequent brain Aβ accumulation.

To our knowledge, only one other longitudinal study (*N* = 32) has considered the relationship between sleep characteristics and brain Aβ accumulation.^4^ Our results, from a larger, sex‐balanced cohort, using a self‐reported sleep measure, extend those previously reported by Winer et al.,^4^ in which an objective measurement of sleep was used. Consistent with our results, Winer et al. found that longer sleep duration predicted slower subsequent brain Aβ accumulation, though this relationship did not survive correction for multiple comparisons in either Winer et al.’s study or the current study.^4^ Winer et al. also reported that poorer sleep efficiency had a trend‐level association with faster accumulation of brain Aβ (r = 0.35, *P* = 0.05)^4^; this relationship did not survive correction for multiple comparisons in the present study when the continuous self‐reported sleep efficiency variable was considered. Additionally, Winer et al. found the rate of brain Aβ accumulation to be predicted by the proportion of non‐rapid eye movement slow wave sleep (SWS) below 1 Hz.^4^ Notably, SWS has been found to play an important role in enhancing brain Aβ clearance via the glymphatic system.^5^, ^8^, ^46^, ^47^, ^48^ While the present study did not include polysomnographic measurement and therefore cannot report on proportion of SWS, our findings of short sleep duration and/or poor sleep efficiency associated with increased rate of brain Aβ accumulation may reflect less opportunity for SWS.

Of note, Winer et al.^4^ did not consider the effect of *APOE* ε4 carriage on the relationship between sleep and brain Aβ accumulation. In line with our expectations that the effects of poor sleep would be magnified in carriers of the *APOE* ε4 allele, we found that sleep duration < 6 hours was associated with a positive slope of brain Aβ accumulation in *APOE* ε4 carriers, but not in the whole sample or in *APOE* ε4 non‐carriers. However, counter to our expectations, we observed a significant relationship between poor sleep efficiency (< 65%) and the subsequent positive slope of brain Aβ accumulation in *APOE* ε4 non‐carriers, but not in carriers. Past research reporting the effect of *APOE* genotype on the relationship between sleep and brain Aβ burden has been scant and inconsistent. Hwang et al.^33^ found, in cross‐sectional analysis of CU older adults from Korea, that *APOE* ε4 carriage moderated the relationship between the sleep–wake cycle and brain Aβ deposition. However, Brown et al.,^3^ using cross‐sectional data from the AIBL Study, reported that *APOE* ε4 carriage did not moderate the sleep onset latency–brain Aβ burden relationship. Further evidence from AIBL suggests that the relationship between sleep and brain Aβ accumulation may involve a combination of genetic factors. Notably, in this cohort, variants of the Aquaporin‐4 gene (*AQP4*), which encodes a key component of the glymphatic system, were found in cross‐sectional analysis to moderate the sleep onset latency–brain Aβ burden relationship.^16^ Further longitudinal analysis is required to investigate the effect of *AQP4* in combination with *APOE* on sleep and brain Aβ accumulation. Nevertheless, as the current study illustrates (Figure 3), poor sleep efficiency provides a potential route via which those at lower genetic risk (*APOE* ε4 non‐carriers) can progress to high brain Aβ burden and subsequent increased risk of AD. Moreover, *APOE* ε4 non‐carriers with < 6 hours’ sleep duration, or *APOE* ε4 carriers with < 65% sleep efficiency (Figures 2 and 3) had greater brain Aβ load at baseline, which remained higher at follow‐up, though the rate of accumulation did not differ across sleep duration or efficiency categories. Future research is required to understand at which stage of life this difference in brain Aβ burden begins to manifest. Such information may inform when sleep interventions aimed at improving short sleep duration or poor sleep efficiency, ideally targeted to *APOE* genotype, may be of the greatest benefit with regard to slowing brain Aβ accumulation and thus maintaining brain health.

With respect to the results of the current study, it is possible that *APOE* ε4 carriers may have overestimated their self‐reported sleep quality. We found that *APOE* ε4 non‐carriers reported significantly shorter sleep duration and had a significantly higher proportion of “poor sleepers” compared to *APOE* ε4 carriers. However, a study by Drogos et al.,^49^ which reported poorer self‐reported sleep quality in *APOE* ε4 non‐carriers, found that this relationship reversed when using objective measures. The authors suggested that in *APOE* ε4 carriers objective changes in sleep quality may precede subjective sleep complaints.^49^ While the current study tried to limit the impact of recall bias by including participants who were CU at the time of self‐report sleep assessment, it is possible that discrepancy between subjective and objective measures of sleep is an important biomarker of brain Aβ burden, and prospective cognitive decline; future studies investigating this hypothesis could prove informative.

Past cross‐sectional studies have reported a relationship between measures of suboptimal sleep quality (defined based on multi‐question questionnaires)^6^, ^7^, ^19^ and higher PET‐measured brain Aβ burden. While we found that for *APOE* ε4 carriers self‐reported “poor” sleep quality (from a single Likert scale response) was nominally associated with faster brain Aβ accumulation, but not in the whole sample or in *APOE* ε4 non‐carriers, this relationship did not survive correction for multiple comparisons. Additionally, we found that PSQI global score, a measure of overall sleep quality, while highly correlated with sleep duration and efficiency, was not associated with the rate of brain Aβ accumulation in any group. Moreover, while prior research has reported a cross‐sectional relationship between longer sleep onset latency and higher brain Aβ burden, we found that in the current study sleep onset latency was not associated with the rate of brain Aβ accumulation.^3^, ^7^, ^20^ However, consistent with past research, longer sleep onset latency was associated with higher brain Aβ at baseline (and higher Aβ burden over time in the current study), compared to shorter sleep onset latency, particularly in the *APOE* ε4 carrier group (Figure S2), though the main effects for these results did not survive correction for multiple comparisons. Given that brain Aβ accumulation is a protracted process^50^ it is possible that the duration of the current study was insufficient to observe associations among PSQI global score, self‐reported sleep quality, and sleep onset latency and rate of brain Aβ increase. It is also conceivable that the characteristics of longer sleep onset latency and poor overall sleep quality make a greater contribution to the rate of brain Aβ accumulation prior to older adulthood. Future research is warranted to address these knowledge gaps.

The novel findings of this study, conducted within a large sex‐balanced cohort of older adults, suggest that markers of poor sleep measured by the PSQI (an accessible, cost‐effective, simple‐to‐administer tool), may act as biomarkers for brain Aβ accumulation. Nevertheless, these findings are not without limitations. Specifically, collection of baseline sleep data and brain Aβ PET imaging were not completed on the same day. However, the slow process of brain Aβ deposition occurs over many years,^50^ while sleep habits are typically chronic in the age group studied. The protracted process of brain Aβ deposition may also mean that the median follow‐up time of 4.3 years was insufficient to observe an effect for all sleep variables. Additionally, while diagnosed obstructive sleep apnea (OSA) represents an exclusion criterion for the AIBL Study, it is possible that some participants have undiagnosed OSA: this is relevant as OSA has previously been associated with higher brain Aβ burden.^51^ Finally, the current study used a self‐report sleep measure, namely the PSQI. While the study included participants who were CU at the time of sleep assessment to circumvent recall bias due to objective memory impairment, self‐report sleep measures still rely on the accuracy of the participant, and, unlike polysomnography, do not provide important information on sleep microarchitecture. Nonetheless, the PSQI has been widely adopted and found to be a valid, reliable measure with good internal consistency for use in older adults, while offering the benefit of greater clinical utility and cost effectiveness.^26^, ^27^

In summary, our findings demonstrate that self‐reported sleep duration < 6 hours in *APOE* ε4 carriers, and sleep efficiency < 65% in the whole cohort and *APOE* ε4 non‐carriers, was associated with faster accumulation of brain Aβ. This study requires validation in independent samples, and extension to mid‐life cohorts to further elucidate these relationships. Future intervention studies, using both objective and self‐report sleep measures are required to determine whether improvements in poor sleep lead to a slowing of the rate of brain Aβ deposition, and to aid our understanding of who may benefit most from such interventions in the context of brain health.

## CONFLICT OF INTEREST STATEMENT

L.N.P., B.M.B., K.R.S., J.D.D., V.D., M.W., H.R.S., S.L.G., R.S.B., S.M.L., K.T., and S.R.R.S. report no disclosures. V.L.V. is and has been a consultant or paid speaker at sponsored conference sessions for Eli Lilly, Life Molecular Imaging, ACE Barcelona, and IXICO. P.M. is a full‐time employee of Cogstate Ltd. C.L.M. is an advisor to Prana Biotechnology Ltd and a consultant to Eli Lilly. C.C.R. has served on scientific advisory boards for Bayer Pharma, Elan Corporation, GE Healthcare, and AstraZeneca; has received speaker honoraria from Bayer Pharma and GE Healthcare; and has received research support from Bayer Pharma, GE Healthcare, Piramal Lifesciences, and Avid Radiopharmaceuticals. R.N.M. is founder of, and owns stock in, Alzhyme, and is a co‐founder of the KaRa Institute of Neurological Diseases. Author disclosures are available in the supporting information

## CONSENT STATEMENT

Prior to undergoing any study procedures or assessments, all individuals provided written informed consent approved by the institutional ethics committees of Austin Health, St. Vincent's Health, Hollywood Private Hospital (now Ramsay Health Care), Murdoch University, and Edith Cowan University.

## Supporting information

Supplemental Information.

Supplemental Information.
