# Supplementary material for: Suboptimal self‐reported sleep efficiency and duration are associated with faster accumulation of brain amyloid beta in cognitively unimpaired older adults
Source: Alzheimers Dement (Amst). 2024 Apr 22;16(2):e12579. doi: 10.1002/dad2.12579 (PMC11033837; doi:10.1002/dad2.12579)
Supplement: Supplementary file 2 — Supplemental Information. [file DAD2-16-e12579-s001.docx]

**Supplementary Statistical Methods**

As non-normality and heteroscedasticity violate assumptions of linear regression modelling and reduce the precision of coefficient estimates (1), the robustness of the model was tested using three Winsorising transformations (80%, 90% and 95%) (2). Increasing the level of Winsorising increasingly reduced heteroscedasticity but did not materially alter the model's results. However, it also generated ranges of Centiloid values that were not representative of the progressive nature of preclinical Alzheimer's disease. Given these findings, the most appropriate transformation proved to be the 95% Winsorised transformation.

While Pearson’s r indicated that sleep duration, sleep efficiency, and Pittsburgh Sleep Quality Index (PSQI) global scores were highly correlated, the correlation coefficients did not exceed 0.90, there was no perfect multicollinearity present (3). The model of best fit was determined based on Akaike Information Criterion (AIC), where the AIC for model selected has substantial support (≤ 2 relative to minimum AIC) (4). This model included random slopes, random intercepts, interaction effects for time and *APOE* ε4 allele carriage, to model against the 95% Winsorised Centiloid value. Covariates excluded through this process included an age-sex interaction, cardiovascular risk score, body mass index (BMI), depressive symptomology (Geriatric Depression Scale; GDS), education category (≤ 12 years / > 12 years) and the use of sleep-related medications.

**Supplementary Section: Table S1.** **Categorical sleep variable levels, referents, and category response frequencies**

| **Categorical sleep models and levels** | **Whole Cohort**  **(*N* = 189)** | | ***APOE ε*4**  **Non-Carriers^c^**  **(*N =* 137)** | | ***APOE ε*4**  **Carriers**  **(*N =* 52*)*** |
| --- | --- | --- | --- | --- | --- |
| **Sleep duration category**  (Drawn from PSQI Question 4, self-report hours of actual sleep)  < 6 hours sleep duration (short)  6-8 hours sleep duration (normal)*  > 8 hours sleep duration (long) | 27  144  18 | | 21  104  12 | | 6  40  6 |
| **Sleep efficiency category**  (Based on PSQI-determined sleep efficiency, which represents the percentage of time in bed spent asleep)  < 65% sleep efficiency (very poor)  65-85% sleep efficiency (suboptimal)  > 85% sleep efficiency (optimal)* | 25  85  79 | | 18  66  53 | | 7  19  26 |
| **Sleep onset latency category**  (Drawn from PSQI Question 2, self-reported minutes of sleep onset latency)  < 30 minutes sleep onset latency (optimal)*  30-59 minutes sleep onset latency (suboptimal)  60+ minutes sleep onset latency (very poor) | 137  36  16 | | 96  27  14 | | 41  9  2 |
| **Sleep disturbance category**  (Drawn from PSQI sleep disturbance component score)  Mild disturbance (component score 0 or 1)*  Moderate / Severe disturbance (component score 2 or 3) | 107  82 | | 74  63 | | 33  19 |
| **Sleep quality category**  (Drawn from PSQI Question 6, self-reported sleep quality)  ‘Very good’*  ‘Fairly good’  ‘Bad’ (response of either “fairly bad” or “very bad”) | 56  104  29 | | 38  77  22 | | 18  27  7 |
|  |  | |  | |  |
| *Referent level of categorical variable  Abbreviations: PSQI, Pittsburgh Sleep Quality Index. | |  | |  |  |

**Supplementary Results**

**Supplementary Table S2. Results of linear mixed models examining the associations between total sleep time, sleep duration category^a^, sleep efficiency, sleep efficiency category^a^, sleep onset latency, sleep onset latency category^a^, Sleep quality category^a^, sleep disturbance category^a^ and PSQI global score, and the slope of brain Aβ-amyloid burden in Centiloid, over time^b^**

| \|  \| **Whole Cohort**  **(*N* = 189)** \| \| ***APOE ε*4 Non-Carriers^c^**  **(*N =* 137)** \| \| ***APOE ε*4 Carriers**  **(*N =* 52*)*** \| \| \| --- \| --- \| --- \| --- \| --- \| --- \| --- \| \| ***Models* / Predictors** \| **β** \| ***p-value*** \| **β** \| ***p-value*** \| **β** \| ***p-value*** \| \| Total sleep time (hr) * Time * *ε*4 \| 0.17 ± 0.46 \| 0.709 \| - \| - \| - \| - \| \| Total sleep time (hr) * Time \| -0.67 ± 0.26 \| **0.012** \| -0.66 ± 0.25 \| **0.010** \| -0.51 ± 0.41 \| 0.218 \| \| Total sleep time \| -2.05 ± 2.12 \| 0.334 \| -1.71 ± 1.90 \| 0.369 \| -9.17 ± 3.57 \| **0.011** \| \| Duration < 6 hr ^d^ * Time * *ε*4 \| 2.99 ± 1.73 \| 0.085 \| - \| - \| - \| - \| \| Duration < 6 hr ^d^ * Time \| 1.29 ± 0.84 \| 0.124 \| 1.25 ± 0.82 \| 0.130 \| 4.33 ± 1.58 \| **0.007^#^** \| \| Duration < 6 hr ^d^ \| 3.71 ± 6.66 \| 0.578 \| 3.55 ± 5.90 \| 0.547 \| 20.75 ± 14.73 \| 0.161 \| \| Duration > 8 hr ^d^ * Time * *ε*4 \| 1.60 ± 1.85 \| 0.388 \| - \| - \| - \| - \| \| Duration > 8 hr ^d^ * Time \| -1.08 ± 1.05 \| 0.304 \| -1.05 ± 1.04 \| 0.310 \| 0.37 ± 1.60 \| 0.816 \| \| Duration > 8 hr ^d^ \| -14.29 ± 8.53 \| 0.094 \| -13.75 ± 7.56 \| 0.070 \| -15.58 ± 15.07 \| 0.303 \| \| Efficiency * Time * *ε*4 \| 0.03 ± 0.04 \| 0.458 \| - \| - \| - \| - \| \| Efficiency * Time \| -0.05 ± 0.02 \| **0.025** \| -0.05 ± 0.02 \| **0.021** \| -0.02 ± 0.04 \| 0.534 \| \| Efficiency \| -0.16 ± 0.19 \| 0.412 \| -0.10 ± 0.17 \| 0.572 \| -0.58 ± 0.35 \| 0.093 \| \| Efficiency < 65% ^e^ * Time * *ε*4 \| -1.70 ± 1.71 \| 0.320 \| - \| - \| - \| - \| \| Efficiency < 65% ^e^ * Time \| 2.91 ± 0.93 \| **0.002^#^** \| 2.86 ± 0.89 \| **0.001^#^** \| 1.32 ± 1.60 \| 0.410 \| \| Efficiency < 65% ^e^ \| 7.89 ± 7.87 \| 0.317 \| 5.78 ± 6.95 \| 0.406 \| 18.98 ± 15.21 \| 0.214 \| \| Efficiency 65 - 85% ^e^ * Time * *ε*4 \| 2.14 ± 1.18 \| 0.070 \| - \| - \| - \| - \| \| Efficiency 65 - 85% ^e^ *Time \| -1.00 ± 0.63 \| 0.111 \| -0.98 ± 0.59 \| 0.099 \| 1.15 ± 1.12 \| 0.307 \| \| Efficiency 65 - 85% ^e^ \| -1.90 ± 5.24 \| 0.717 \| -2.66 ± 4.61 \| 0.565 \| 6.74 ± 10.69 \| 0.529 \| \| Latency (min) * Time * *ε*4 \| -0.00 ± 0.03 \| 0.956 \| - \| - \| - \| - \| \| Latency (min) * Time \| 0.02 ± 0.01 \| 0.243 \| 0.02 ± 0.01 \| 0.227 \| 0.01 ± 0.03 \| 0.665 \| \| Latency (min) \| 0.10 ± 0.11 \| 0.371 \| 0.08 ± 0.10 \| 0.411 \| 0.42 ± 0.29 \| 0.157 \| \| Latency 30 - 59 min ^f^ * Time * *ε*4 \| 1.38 ± 1.51 \| 0.362 \| - \| - \| - \| - \| \| Latency 30 - 59 min ^f^ * Time \| 0.03 ± 0.78 \| 0.966 \| 0.00 ± 0.76 \| 0.997 \| 1.45 ± 1.38 \| 0.297 \| \| Latency 30 - 59 min ^f^ \| 1.68 ± 6.19 \| 0.786 \| 1.09 ± 5.48 \| 0.842 \| -0.29 ± 12.28 \| 0.981 \| \| Latency 60 min+ ^f^ * Time * *ε*4 \| -0.44 ± 2.86 \| 0.877 \| - \| - \| - \| - \| \| Latency 60 min+ ^f^ * Time \| 0.46 ± 1.02 \| 0.651 \| 0.48 ± 0.99 \| 0.629 \| -0.02 ± 2.87 \| 0.993 \| \| Latency 60 min+ ^f^ \| 7.22 ± 8.24 \| 0.381 \| 6.21 ± 7.33 \| 0.397 \| 46.77 ± 24.61 \| 0.059 \| \| Fairly good quality ^g^ * Time * *ε*4 \| 0.38 ± 1.27 \| 0.763 \| - \| - \| - \| - \| \| Fairly good quality ^g^ * Time \| 0.56 ± 0.70 \| 0.417 \| 0.57 ± 0.68 \| 0.404 \| 0.94 ± 1.11 \| 0.397 \| \| Fairly good quality ^g^ \| -6.08 ± 5.60 \| 0.278 \| -6.13 ± 4.92 \| 0.214 \| 5.12 ± 10.40 \| 0.623 \| \| Bad quality * Time ^g^ * *ε*4 \| 2.72 ± 1.83 \| 0.137 \| - \| - \| - \| - \| \| Bad quality ^g^ * Time \| 0.67 ± 0.95 \| 0.479 \| 0.69 ± 0.93 \| 0.460 \| 3.42 ± 1.64 \| **0.038** \| \| Bad quality ^g^ \| -3.76 ± 7.63 \| 0.622 \| -4.38 ± 6.72 \| 0.515 \| 24.80 ± 15.21 \| 0.105 \| \| Moderate / severe disturbance ^h^ * Time * *ε*4 \| 2.35 ± 1.17 \| **0.046** \| - \| - \| - \| - \| \| Moderate / severe disturbance ^h^ * Time \| -0.43 ± 0.61 \| 0.474 \| -0.43 ± 0.59 \| 0.473 \| 1.96 ± 1.06 \| 0.065 \| \| Moderate / severe disturbance ^h^ \| -4.96 ± 4.92 \| 0.314 \| -5.96 ± 4.28 \| 0.165 \| -4.24 ± 9.96 \| 0.671 \| \| PSQI global score * Time * *ε*4 \| 0.09 ± 0.16 \| 0.577 \| - \| - \| - \| - \| \| PSQI global score * Time \| 0.13 ± 0.09 \| 0.155 \| 0.12 ± 0.09 \| 0.148 \| 0.23 ± 0.13 \| 0.072 \| \| PSQI global score \| -0.26 ± 0.72 \| 0.722 \| -0.47 ± 0.10 \| 0.059 \| 0.23 ± 0.64 \| 0.460 \| \| Models include a sleep measure, *APOE* ε4 allele carrier status (+/-), time from baseline PET scan until final PET scan, baseline age and sex as main effects and three-way sleep**APOE* ε4 status*Centiloid interaction. Beta coefficients (β) ± SE from the LMM are shown, with bold indicating nominal significance (*p* < 0.05) and ^#^ indicating significance following Bonferroni correction (*p* < 0.008). ^a^ See Supplementary Table S1 for description of categorical variables. ^b^ Median follow up period of 51.55 months (MAD = 13.01). ^c^ Absence of any ε4 alleles. ^d^ Compared to 6 - 8 hours sleep duration. ^e^ Compared to > 85% sleep efficiency. ^f^ Compared to < 30 minutes sleep onset latency. ^g^ Compared to “very good” sleep quality. ^h^ Compared with “mild disturbance”.  Abbreviations: *APOE*, apolipoprotein E; Efficiency, Sleep efficiency; hr, hours; Latency, Sleep onset latency; LMM, linear mixed model; MAD, median absolute deviation; min, minutes; PET, positron emission tomography; PSQI, Pittsburgh Sleep Quality Index; SE, standard error. \| \| \| \| \| \| \| |
| --- | --- | --- | --- | --- | --- | --- | --- | --- | --- | --- | --- | --- | --- | --- | --- | --- | --- | --- | --- | --- | --- | --- | --- | --- | --- | --- | --- | --- | --- | --- | --- | --- | --- | --- | --- | --- | --- | --- | --- | --- | --- | --- | --- | --- | --- | --- | --- | --- | --- | --- | --- | --- | --- | --- | --- | --- | --- | --- | --- | --- | --- | --- | --- | --- | --- | --- | --- | --- | --- | --- | --- | --- | --- | --- | --- | --- | --- | --- | --- | --- | --- | --- | --- | --- | --- | --- | --- | --- | --- | --- | --- | --- | --- | --- | --- | --- | --- | --- | --- | --- | --- | --- | --- | --- | --- | --- | --- | --- | --- | --- | --- | --- | --- | --- | --- | --- | --- | --- | --- | --- | --- | --- | --- | --- | --- | --- | --- | --- | --- | --- | --- | --- | --- | --- | --- | --- | --- | --- | --- | --- | --- | --- | --- | --- | --- | --- | --- | --- | --- | --- | --- | --- | --- | --- | --- | --- | --- | --- | --- | --- | --- | --- | --- | --- | --- | --- | --- | --- | --- | --- | --- | --- | --- | --- | --- | --- | --- | --- | --- | --- | --- | --- | --- | --- | --- | --- | --- | --- | --- | --- | --- | --- | --- | --- | --- | --- | --- | --- | --- | --- | --- | --- | --- | --- | --- | --- | --- | --- | --- | --- | --- | --- | --- | --- | --- | --- | --- | --- | --- | --- | --- | --- | --- | --- | --- | --- | --- | --- | --- | --- | --- | --- | --- | --- | --- | --- | --- | --- | --- | --- | --- | --- | --- | --- | --- | --- | --- | --- | --- | --- | --- | --- | --- | --- | --- | --- | --- | --- | --- | --- | --- | --- | --- | --- | --- | --- | --- | --- | --- | --- | --- | --- | --- | --- | --- | --- | --- | --- | --- | --- | --- | --- | --- | --- | --- | --- | --- | --- | --- | --- | --- | --- | --- | --- |

**
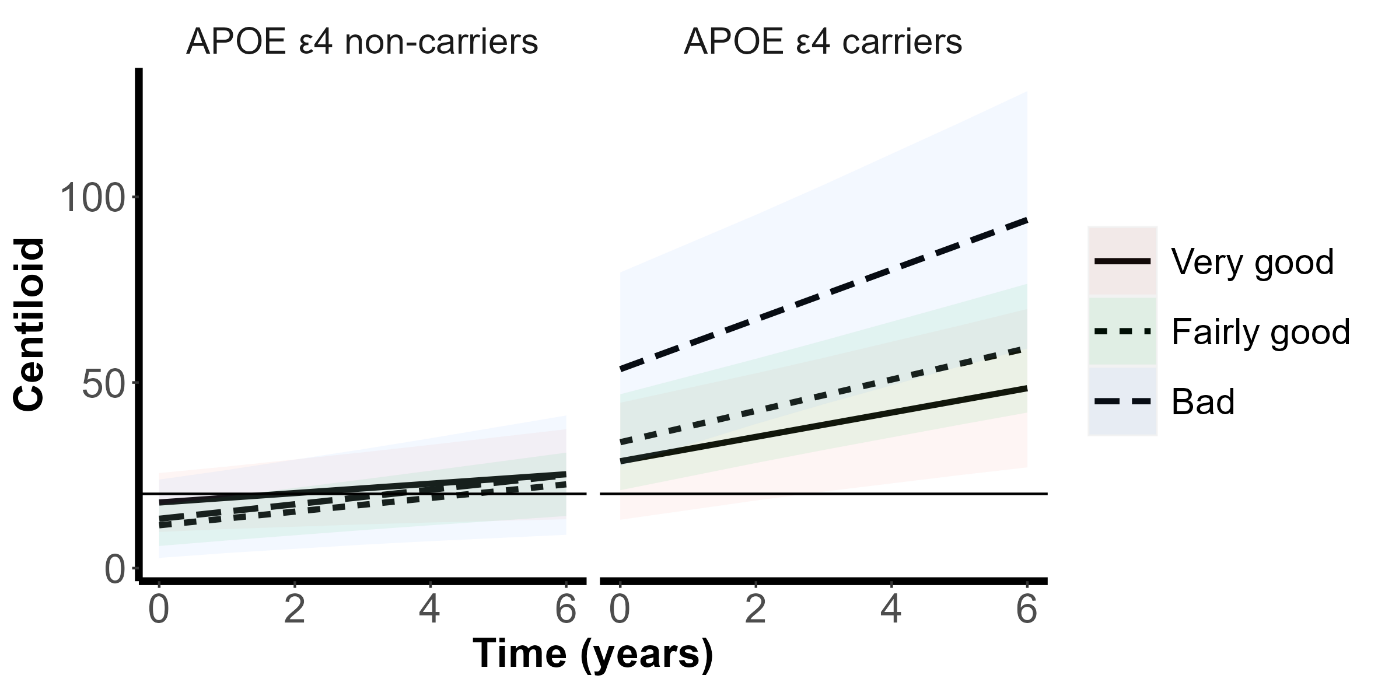
**

**Figure S1. Plots for the relationship between baseline sleep quality category and the trajectory of brain Aβ-amyloid burden, in cognitively unimpaired *APOE* ε4 allele non-carriers (left) and carriers (right).** Brain Aβ-amyloid burden is presented as Centiloid value (95% Winsorised). Sleep quality category was derived from PSQI Question 6, self-reported sleep quality, yielding ‘Very good’, ‘Fairly good’, or ‘Bad’ (response of either “fairly bad” or “very bad”) groupings. The horizontal line, at a Centiloid value of 20, represents the threshold for high brain Aβ-amyloid burden. Coloured bands represent 95% confidence intervals. “Bad sleep” was associated with greater brain Aβ-amyloid burden at baseline and predicted a steeper slope of brain Aβ-amyloid accumulation in *APOE* ε4 allele carriers. Abbreviations: *APOE*, Apolipoprotein E gene; PSQI, Pittsburgh Sleep Quality Index.


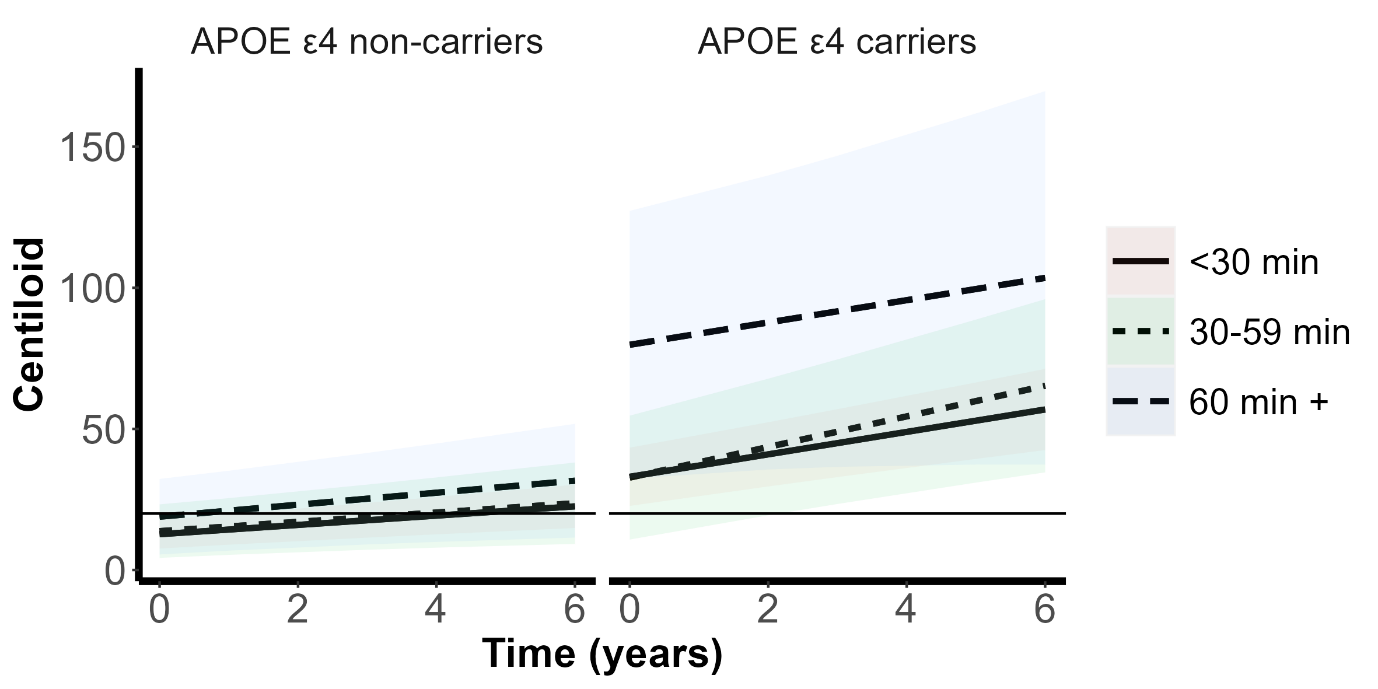


**Figure S2. Plots for the relationship between baseline sleep onset latency category and the trajectory of brain Aβ-amyloid burden, in cognitively unimpaired *APOE* ε4 allele non-carriers (left) and carriers (right).** Brain Aβ-amyloid burden is presented as Centiloid value (95% Winsorised). Sleep onset latency (time to fall asleep) category was derived from PSQI Question 2, self-reported minutes of sleep onset latency, where < 30 minutes sleep onset latency is optimal, 30-59 minutes is suboptimal, and 60+ minutes is categorised as very poor. The horizontal line, at a Centiloid value of 20, represents the threshold for high brain Aβ-amyloid burden. Coloured bands represent 95% confidence intervals. Sleep onset latency category did not predict the slope of brain Aβ-amyloid accumulation in *APOE* ε4 non-carriers or carriers. Abbreviations: *APOE*, Apolipoprotein E gene; min, minutes; PSQI, Pittsburgh Sleep Quality Index.

**Supplementary Section References**

1. Schielzeth H, Dingemanse NJ, Nakagawa S, Westneat DF, Allegue H, Teplitsky C, et al. Robustness of linear mixed-effects models to violations of distributional assumptions. Methods in Ecology and Evolution. 2020;11(9):1141-52.

2. Ruppert D. Trimming and Winsorization. Wiley StatsRef: Statistics Reference Online. 2014.

3. Tabachnick BG, Fidell LS. Using multivariate statistics. 6th ed. ed. Boston: Pearson Education; 2013.

4. Burnham KP, Anderson DR. Multimodel inference: Understanding AIC and BIC in model selection. Sociological Methods and Research. 2004;33(2):261-304.

5. Team RS. RStudio: Integrated Development Environment for R. Boston, MA: RStudio, PBC; 2021.
